# Unravelling the Relationship: Oral Protozoans, Periodontitis and Systemic Non-Communicable Diseases

**DOI:** 10.21315/mjms-10-2024-808

**Published:** 2025-02-28

**Authors:** Nurin Jazlina Nor Azmi, Suharni Mohamad, Zeehaida Mohamed

**Affiliations:** 1 School of Dental Sciences, Universiti Sains Malaysia, Health Campus, Kelantan, Malaysia; 2 Department of Medical Microbiology and Parasitology, School of Medical Sciences, Universiti Sains Malaysia, Health Campus, Kelantan, Malaysia

**Keywords:** periodontal disease, periodontitis, oral protozoans, Entamoeba gingivalis, Trichomonas tenax, noncommunicable diseases

## Abstract

Oral protozoans, specifically *Entamoeba gingivalis* and *Trichomonas tenax*, have been identified in the oral cavity of individuals with periodontitis, frequently known as gum disease. Periodontitis is characterised by inflammation and degradation of the periodontal tissues and has been associated with the aggravation of systemic noncommunicable diseases. Although the importance of oral protozoans in periodontitis progression is often overlooked, their occurrence in individuals with periodontitis has reportedly been linked to the same modifiable risk factors contributing to numerous systemic noncommunicable diseases (NCDs). This article aims to provide a comprehensive overview of the occurrence patterns and potential connections among oral protozoans, periodontitis, and systemic NCDs while presenting the existing methods for the detection and identification of *E. gingivalis* and *T. tenax*.

## Introduction

Oral health issues are among the most prominent diseases affecting the oral cavity and its surrounding structures. These issues have emerged as critical health concerns across 194 countries, with a global prevalence rate of 45% (1). The World Health Organization stated in its Global Oral Health Status Report for 2022 that approximately 3.5 billion individuals, comprising 50% of the global population, suffer from oral diseases. The reported incidence of oral health issues surpasses the combined incidence of the five most prevalent noncommunicable diseases (NCDs), comprising diabetes, cardiovascular diseases, chronic respiratory diseases, mental disorders, and cancers, by approximately 1 billion cases. Dental caries, severe periodontal disease, and edentulism are the primary oral health issues that impose a substantial burden on human health worldwide (2, 3).

Periodontal disease, frequently known as gum disease, is an inflammatory condition that affects the structures of the periodontium comprising the gums, alveolar bones, and periodontal ligaments. Periodontitis is a severe form of periodontal disease, accounting for > 1 billion reported cases, which is equivalent to 19% of the global adult population (1, 4). The predominant factor affecting the formation of gum pockets, resulting in heightened tooth mobility and ultimately tooth loss. This progression leads to masticatory dysfunction and contributes to speech impairment and alterations in facial appearance, thereby affecting an individual’s self-confidence and overall quality of life (5–7).

The initiation of periodontitis stems from the disruption of the oral microbiota, which comprises various microorganisms, including bacteria, viruses, and protozoans. No single pathogen is solely responsible for the disease; instead, various factors collectively lead to disease development and progression (7–9). Notably, the oral protozoans *Entamoeba gingivalis* and *Trichomonas tenax* have been detected in periodontal pockets and are linked with the progression of periodontitis. The occurrence of these oral protozoans in individuals with periodontitis is associated with the same modifiable risk factors that are common to numerous systemic NCDs, such as poor dietary habits, inadequate oral care, alcohol consumption, and tobacco use (10, 11). Nevertheless, the criticality of these oral protozoans in the progression of periodontitis has been neglected.

The Sustainable Development Goals (SDGs), proposed by the United Nations, represent a strategic framework aimed at achieving the 2030 Agenda, primarily focusing on eradicating poverty and fostering universal peace. Among the 17 SDGs, SDG 3 emphasises attaining and promoting holistic well-being and health for all, focusing on universal health coverage and the reduction of NCDs (12, 13). Common risk factors of periodontitis and systemic NCDs should be highlighted considering their existing interrelations. Preventing oral diseases could play a notable role in averting the exacerbation of NCDs; this preventive approach can reduce the burden of NCDs and align with the pursuit of achieving SDG 3 (12, 14).

This study aimed to unravel the complex interplay between oral protozoans and periodontitis concerning systemic NCDs, specifically diabetes, cardiovascular diseases, chronic respiratory diseases, and cancers. It will analyse occurrence patterns and clarify potential reciprocal interactions among oral protozoans, periodontitis, and systemic NCDs, particularly diabetes, cardiovascular diseases, respiratory diseases, and cancer, primarily focus on associated risk factors. This study will also explore various oral protozoan detection methods. The goal is to gain a deep understanding of these interrelationships and establish the groundwork for interventions and preventive measures through public health initiatives and policy development, thereby facilitating the quest to realise SDG 3.

## Materials and Methods

The online databases PubMed, Scopus, Web of Science, and Google Scholar were used to search for relevant literature using specific keywords. These keywords included oral protozoans, *Trichomonas tenax*, *Entamoeba gingivalis*, periodontitis, periodontal diseases, gum disease, NCDs, systemic diseases, diabetes, cardiovascular diseases, chronic respiratory diseases, and cancers. Multiple searches were conducted using various keyword combinations. Articles published in English; full-text papers; studies that solely addressed the association between oral protozoans, periodontitis, and systemic diseases; and articles involving humans were included in the study.

**Figure 1:**
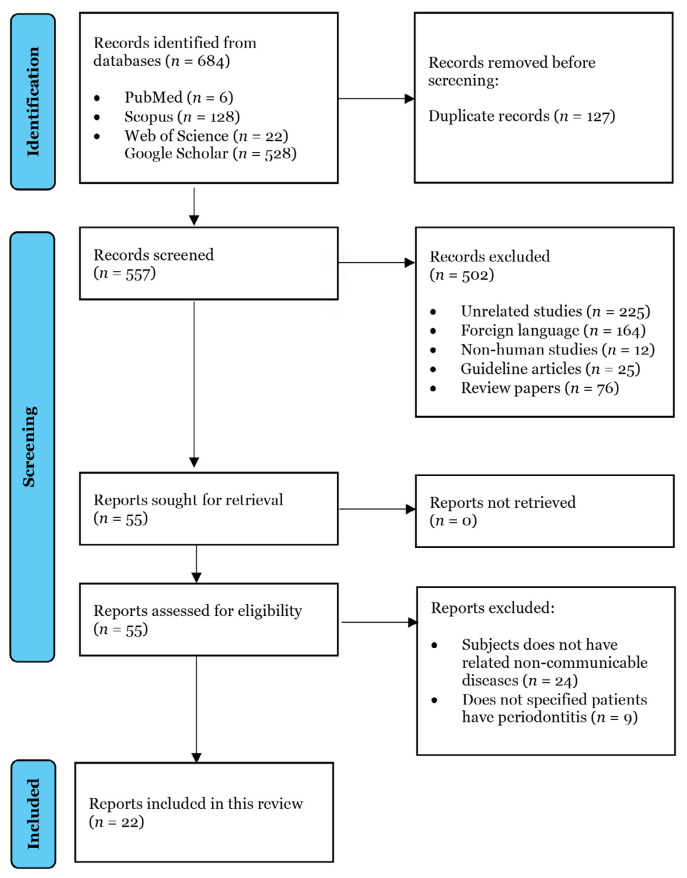
Flow diagram depicting the process of records identification, screening and inclusion in the review according to PRISMA 2020 (36).

The initial search retrieved 684 articles. Paper titles and abstracts of each search result were screened, and after excluding duplicates, nonhuman studies, unrelated research, articles in foreign languages, and papers not available in full-text, 55 articles remained for evaluation. The full-text papers were reviewed and assessed for eligibility. Of these, 22 articles met the inclusion criteria and were included (Figure 1). These articles collectively explored the associations among oral protozoans, periodontitis, and systemic diseases rather than establishing causality. Table 1 summarises the level of evidence for each included article based on the standard levels outlined by the Oxford Centre for Evidence-Based Medicine (15).

## Results and Discussion

### Oral Protozoans, Periodontitis and NCDs

The presence of oral protozoans, known as *E. gingivalis* and *T. tenax*, in the human oral cavity has been observed to augment among individuals with periodontitis, a condition characterised by inflammation and irreversible destruction of the periodontium and alveolar bone surrounding the teeth (37). In a cohort of 80 patients with periodontitis, the prevalence of *E. gingivalis* and *T. tenax* was 88.9% and 25.6%, respectively (30); this highlights the importance of oral protozoans in periodontitis, which has been linked to systemic NCDs (37–40). Oral protozoans can amplify the inflammatory response associated with periodontitis (41), thereby possibly contributing to the worsening of systemic inflammation and aggravating systemic diseases.

Diabetes is a chronic metabolic disorder characterised by prolonged elevation of blood sugar levels because of issues with insulin production or function; this condition has widespread effects on the body and can promote systemic inflammation through various mechanisms, including hyperglycemia, contributing to tissue damage and dysfunction in multiple organs (42, 43).

The prevalence of periodontitis in patients with diabetes was 59.5% (21). Reportedly, the incidence of *E. gingivalis* and *T. tenax* in the oral cavity of patients with diabetes afflicted with periodontitis was 6%–88.1% and 6%–70%, respectively (10, 19, 25, 26, 32–35). Al-Sarhan et al. (27) demonstrated that the prevalence of periodontitis is two times higher among patients with diabetes than among those without diabetes, with a 61% rate of *E. gingivalis* infection. The heightened severity of parasitic infection in patients with diabetes compared with healthy individuals (33.3%) is concerning, considering their compromised ability to regulate blood sugar levels. This creates a favourable environment for bacteria and protozoans to proliferate, subsequently intensifying gum destruction and hindering wound healing, thereby advancing periodontitis (44). Additionally, the incidence of *E. gingivalis* and *T. tenax* was higher among patients with diabetes than those with other chronic diseases at 20% and 18.91%, respectively (11).

Several case reports (16–18, 20, 28, 29, 31), have documented the presence of *T. tenax* in respiratory diseases, particularly empyema, pyopneumothorax, and pulmonary trichomoniasis. Although these conditions are frequently classified as acute rather than chronic respiratory diseases such as asthma, we opted to incorporate these findings in this study because of their potential to develop as complications of underlying respiratory conditions, such as pneumonia (45).

Hypothetically, this flagellated protozoan enters the lower respiratory tract via aspiration from the mouth and the contaminated oropharynx. Its proliferation relies on concomitant anaerobic and aerobic bacterial species that act as food sources for *T. tenax* (17, 18, 20, 29, 31). In an epidemiological study conducted by Al-Quraishi et al. (22), which was consistent with these findings, the prevalence of *T. tenax* and opportunistic pulmonary trichomoniasis infection was 1.99%. Conversely, Fadhil Ali Malaa et al. (11) reported that 6.66% and 5.4% of patients with asthma tested positive for *E. gingivalis* and *T. tenax*, respectively.

Our search yielded limited articles on the incidence of oral protozoans among patients with periodontitis and cancer or cardiovascular diseases. Nevertheless, Al-Zubaidi (24) provided evidence of oral protozoans in patients with carcinoma of the lower alveoli and carcinoma of the hard palate, indicating the presence of *E. gingivalis* and *T. tenax* in 50% of oral carcinoma cases. Cancer and its treatment, including chemotherapy and radiotherapy, may induce various side effects, such as immunological disorders, which may compromise the symbiosis of the oral mucosa and elevate the risk of bacterial, viral, and parasitic infections. Consequently, it can exacerbate gum inflammation and contribute to the deterioration of oral health (46). Fadhil Ali Malaa et al. (11) reported incidences of 6.66% and 5.44% for *E. gingivalis* and *T. tenax* in patients with cancer. However, among patients with heart disease, the prevalence of *E. gingivalis* and *T. tenax* was 10% and 8.1%, respectively. The lack of substantial findings suggests that these cases are rare, possibly nonexistent, or relatively unexplored, implying opportunities for further research and investigation.

### Risk Factors

Individuals with systemic NCD frequently have compromised immune systems, thereby increasing their susceptibility to infections. This heightened vulnerability subsequently accelerates disease progression and adds to the overall burden of patients (11, 39). The presence of oral protozoans among patients with systemic NCDs affected by periodontitis indicate potential interplay between these subjects.

Meabed and Henin (32) observed that modifiable risk factors, such as oral hygiene and tobacco use, influence the prevalence of oral protozoans in patients with diabetes and periodontitis. Their findings indicated that 80% and 77.1% of the patients with periodontitis in the diabetic group with poor oral hygiene tested positive for *E. gingivalis* and *T. tenax*, respectively. These results are consistent with those of Fadhil Ali Malaa et al. (11), who reported that 67% and 71% of patients not practicing oral hygiene had *E. gingivalis* and *T. tenax*, respectively. Inadequate oral hygiene leads to poor oral health, further promoting the growth and multiplication of pathogenic microorganisms within the oral microbiota. These changes create a conducive environment for oral protozoans to proliferate, increasing the risk of gum inflammation and advancing disease progression (19).

The incidence of *E. gingivalis* and *T. tenax* is relatively high among patients with diabetes and periodontitis who smoke, reaching a rate of 79.4%. This association demonstrated a statistically significant correlation (*p* < 0.05), indicating a positive association between smoking and the prevalence of oral protozoans (32). Similarly, Fadhil Ali Malaa et al. (11) noted a higher incidence of *E. gingivalis* (66%) and *T. tenax* (78%) among smokers than among nonsmokers. The use of tobacco disrupts the balance of the oral microbiota, facilitating the colonisation of pathogenic bacteria, and compromising the function of periodontal ligament cells. These effects indirectly encourage the invasion of oral protozoans (47).

In reported case studies of respiratory diseases, patients with empyema, pyopneumothorax, and pulmonary trichomoniasis were predominantly observed to exhibit poor oral hygiene (16–18, 28, 29, 31). Moreover, smoking was identified as a contributing factor in case studies by Cai and Fang (31) and Karigoudar et al. (28).

Research exploring the potential risk factors in patients with periodontitis and systemic NCDs is limited, despite the shared risk factors for periodontitis and systemic diseases. Therefore, emphasising the vital role of oral hygiene and the influence of tobacco use on the oral microbiota is crucial; in turn, this may impact an individual’s overall health and well-being.

### Screening and Diagnostic Approaches

*E. gingivalis* and *T. tenax*, motile protozoans residing in the oral cavity, can be distinguished based on their size, flagella appearance, and the presence of an undulating membrane. *E. gingivalis* typically appears as an ameboid, measuring 12–25 μm in diameter, and moves by extending its pseudopodia. In contrast, *T. tenax* is ovoid or ellipsoidal measuring 12–20 μm long and 5–6 μm wide and is characterised by a flagellum and an undulating membrane (41).

The identification of oral protozoans in patients with periodontitis, diabetes, cancer, and cardiovascular diseases can be performed using various specimens, such as saliva, dental plaque, dental calculus, mouth scrapings, oral swabs, cough samples, extracted teeth, and mouth fluid obtained through rinsing with mouthwash. The rate of parasite detection varied across different sample types in all reported studies, primarily owing to variations in sample sizes. Mouth scraping exhibited the highest rate of detection for *E. gingivalis* (88.13%) (33), whereas dental plaque and a mixture of saliva with calculus had the highest detection rate for *T. tenax* (70%) (32). Piekarczyk et al. (19) used dental plaque samples to detect oral protozoans, resulting in a comparatively low detection rate of 4%. Conversely, other studies have reported higher detection rates—26.08%–80% for *E. gingivalis* and 12%–70% for *T. tenax* (11, 30, 32).

In patients with respiratory diseases presenting signs of pleural effusion, diagnostic sampling typically involves pleural fluid obtained via thoracentesis, a procedure aimed at draining fluid from the pleural cavity. This approach is necessary because excessive pleural fluid accumulation can impair the respiratory system (48). The discovery of *T. tenax* in these reported case studies is incidental while diagnosing, underscoring its relevance as a source for diagnostic testing.

The current detection methods for oral protozoans are diverse and range from conventional microscopy and cell culture to molecular techniques such as polymerase chain reaction (PCR) and next-generation sequencing (NGS). Microscopic examination, facilitated by wet-mount or staining techniques such as Giemsa, hematoxylin and eosin, trichrome, and Papanicolaou staining, was frequently favoured by the studies included herein, with 18 articles employing this method. Oral protozoans were identified by observing their mobility and morphological characteristics using light or phase-contrast microscopy. This method is cost-effective and provides quick results. However, the reliability of the microscopic approach heavily relies on the expertise and skills of the examiner, in addition to the lag time between sampling and examination (30), which may inadvertently introduce biases in the interpretation of results and reduce accuracy.

Piekarczyk et al. (19) and Meabed and Henin (32) developed in vitro culture techniques that can increase the abundance of oral protozoans in a given sample. Specifically, oral protozoans were cultured from dental plaque samples and a mixture of saliva with calculus using Locke’s egg medium and Diamond’s trypticase-yeast-maltose (TYM) medium; this resulted in a detection rate of 62.5% and 53% for *E. gingivalis* and *T. tenax*, respectively (32). However, in case reports presenting the incidence of *T. tenax* with respiratory diseases, attempts to cultivate the protozoan were unsuccessful (16, 17, 29). This challenge in cultivating *T. tenax* can be attributed to its high sensitivity to environmental changes, such as fluctuations in pH and temperature (10), along with the substantial risk of contamination from host components, which may influence the cultivation process (49, 50).

Herein, several studies utilised molecular approaches, which are considerably the most accurate methods for detecting oral protozoans due to their high sensitivity and specificity. Wu et al. (29) and Cai and Fang (31) employed NGS in their investigations, whereas Al-Sarhan et al. (27), Bellanger et al. (20), Bracamonte-Wolf et al. (10), Dybicz et al. (23), and Yaseen et al. (30) performed PCR analysis to determine the presence of oral protozoans. In most studies, genomic DNA extraction from the collected samples was performed either using a ready-made kit or by outsourcing to a company. However, Bracamonte-Wolf et al. (10) used the phenol-chloroform method to extract DNA.

The primers used in the PCR analyses varied according to the target regions (Table 2). Al-Sarhan et al. (27) and Yaseen et al. (30) amplified the target regions of *E. gingivalis* using 18S-SSU rDNA and 18S rRNA, with detection rates of 52% and 71.7%, respectively. Meanwhile, for *T. tenax*, the primers employed included the beta-tubulin gene, 18S rRNA, 5.8S rRNA, and the internal transcribed spacer flanking regions ITS1 and ITS2 (10, 20, 23, 30). PCR analysis revealed varying detection rates of *T. tenax*, with the highest rate achieved using the beta-tubulin gene primer (53%), and the lowest rate obtained by amplifying the 5.8S rRNA and flanking regions at 14.1% (10, 23).

Employing molecular techniques such as NGS and PCR for the specific targeting and rapid identification of oral protozoans is more practical and time-efficient than traditional methods of microscopic examination and culture (10, 20, 31). This approach remarkably enhances the efficiency of detecting oral protozoans in patients with periodontitis and systemic comorbidities, leading to reduced diagnostic time and improved diagnostic accuracy.

## Conclusion

The remarkably high prevalence of oral protozoans among individuals with periodontitis and systemic NCDs warrants attention. Addressing oral health as an integral aspect of overall health and well-being is crucial, considering previous research findings have highlighted the presence of oral protozoans causing periodontitis in individuals with systemic NCDs. Moreover, shared risk factors suggest that periodontitis could serve as a mediator between oral protozoans and systemic NCDs, potentially exacerbating underlying systemic conditions. The potential roles of periodontitis and oral protozoans in the worsening of systemic NCDs warrant further investigation. To achieve SDG 3 and reduce global health disparities, oral health must be integrated into broader public health initiatives and policy development. This integrated approach is essential for effectively managing NCDs and promoting overall health equity.

## Figures and Tables

**Table 1:** Level of evidence for each included article

Authors	Year published	Study type	Country	Level of evidence
Hersh (16)	1985	Case report	USA	VI
Shiota et al. (17)	1998	Case report	Japan	VI
Lewis et al. (18)	2003	Case report	USA	VI
Piekarczyk et al. (19)	2003	Cross-sectional study	USA	V
Bellanger et al. (20)	2008	Case report	France	VI
Rajhans et al. (21)	2011	Cross-sectional study	India	V
Al-Quraishi et al. (22)	2015	Cross-sectional study	Iraq	V
Dybicz et al. (23)	2018	Cross-sectional study	Poland	V
Al-Zubaidi (24)	2019	Case-control study	Iraq	IV
Bracamonte-Wolf et al. (10)	2019	Cross-sectional study	Chile	V
Hassan et al. (25)	2019	Case-control	Egypt	IV
Mohammed and Alwaaly (26)	2019	Cross-sectional study	Iraq	V
Al-Sarhan et al. (27)	2021	Case-control study	Iraq	IV
Karigoudar et al. (28)	2021	Case report	India	VI
Wu et al. (29)	2021	Case report	China	VI
Yaseen et al. (30)	2021	Cohort	Jordan	III
Cai and Fang (31)	2022	Case report	China	VI
Fadhil Ali Malaa et al. (11)	2022	Cross-sectional study	Iraq	V
Meabed and Henin (32)	2022	Case-control study	Egypt	IV
Mohammed Jabbary and Hamad (33)	2022	Cross-sectional study	Iraq	V
Nuaimi (34)	2022	Cross-sectional study	Iraq	V
Nuaimi et al. (35)	2022	Cross-sectional study	Iraq	V

**Table 2:** PCR analysis of *E. gingivalis* and *T. tenax*.

*E. gingivalis*				
Gene target	Primers	Sample type	Detection rate among patients with NCDs	Reference
18S-SSU rDNA	Forward: F_GAATAGGCGCGCATTTCGAACAGG Reverse: R_2TCCCACTAGTAAGGTACTACTC	Gum swabs, gum pockets, saliva and extracted teeth	61%	Al-Sarhan et al. (27)
18S rRNA	Forward: 5′-AGGAATGAACGGAACGTACA-3′ Reverse: 5′-CCATTTCCTTCTTCTATTGTTTMAC-3′	Mixture of saliva and dental plaque	78.6%	Yaseen et al. (30)

***T. tenax***				

**Gene target**	**Primers**	**Sample type**	**Detection rate among patients with NCDs**	**Reference**

5.8S rRNA, ITS1 and ITS2	Not stated	Pleural fluid, sputum, and bronchoalveolar fluid	Case study	Bellanger et al. (20)
Beta-tubulin gene	Tt β-tub (sense): 5′-ATACTCTATCGTCCCATCTC-3′ Tt β-tub (antisense): 5′-GCCATCATGTTCTTGTTATCG-3′.	Dental plaque	23.3%	Bracamonte-Wolf et al. (10)
ITS1-5.8S rRNA-ITS2	T1: 5’GAGAAGTCGTAACAAGGTAACG-3’ T2: 5’-ATGCTTCAGTTCAGCGGGTCT-3’	Mouth fluid	14.1%	Dybicz et al. (23)
18S rRNA	PT3 forward: 5′-AGTTCCATCGATGCCATTC-3′ PT7 reverse: 5′-GCATCTAAGGACTTAGACG-3′	Mixture of saliva and dental plaque	None out of 14 diabetic patients Overall prevalence was 27%	Yaseen et al. (30)

## Abbreviations

DNA: deoxyribonucleic acid
S-SSU rDNA: small subunit ribosomal DNA
S rRNA: small regulatory ribonucleic acid
ITS: internal transcribed spacer
